# Does Platelet-Rich Fibrin Enhance the Early Angiogenetic Potential of Different Bone Substitute Materials? An In Vitro and In Vivo Analysis

**DOI:** 10.3390/biomedicines9010061

**Published:** 2021-01-10

**Authors:** Sebastian Blatt, Daniel G. E. Thiem, Andreas Pabst, Bilal Al-Nawas, Peer W. Kämmerer

**Affiliations:** 1 Department of Oral and Maxillofacial Surgery, University Medical Center, Augustusplatz 2, 55131 Mainz, Germany; daniel.thiem@unimedizin-mainz.de (D.G.E.T.); al-nawas@uni-mainz.de (B.A.-N.); Peer.Kaemmerer@unimedizin-mainz.de (P.W.K.); 2 Platform for Biomaterial Research, BiomaTiCS Group, University Medical Center, Langenbeckstrasse 1, 55131 Mainz, Germany; 3 Department of Oral and Maxillofacial Surgery, Federal Armed Forces Hospital, Rübenacherstr. 170, 56072 Koblenz, Germany; andipabst@me.com

**Keywords:** angiogenesis, platelet-rich fibrin, tissue engineering, osteogenesis

## Abstract

The impaired angiogenic potential of bone substitute materials (BSMs) may limit regenerative processes. Therefore, changes in the angiogenetic properties of different BSMs in combination with platelet-rich fibrin (PRF) in comparison to PRF alone, as well as to native BSMs, were analyzed in vitro and in vivo to evaluate possible clinical application. In vitro, four BSMs of different origins (allogeneic, alloplastic, and xenogeneic) were biofunctionalized with PRF and compared to PRF in terms of platelet interaction and growth factor release (vascular endothelial growth factor (VEGF), tissue growth factor ß (TGFß) and platelet-derived growth factor (PDGF)) after 15 min. To visualize initial cell–cell interactions, SEM was performed. In vivo, all BSMs (±PRF) were analyzed after 24 h for new-formed vessels using a chorioallantoic membrane (CAM) assay. Especially for alloplastic BSMs, the addition of PRF led to a significant consumption of platelets (*p* = 0.05). PDGF expression significantly decreased in comparison to PRF alone (all BSMs: *p* < 0.013). SEM showed the close spatial relation of each BSM and PRF. In vivo, PRF had a significant positive pro-angiogenic influence in combination with alloplastic (*p* = 0.007) and xenogeneic materials (*p* = 0.015) in comparison to the native BSMs. For bio-activated xenogeneic BSMs, the branching points were also significantly increased (*p* = 0.005). Finally, vessel formation was increased for BSMs and PRF in comparison to the native control (allogeneic: *p* = 0.046; alloplastic: *p* = 0.046; and xenogeneic: *p* = 0.050). An early enhancement of angiogenetic properties was demonstrated when combining BSMs with PRF in vitro and led to upregulated vessel formation in vivo. Thus, the use of BSMs in combination with PRF may trigger bony regeneration in clinical approaches.

## 1. Introduction

Bone substitute materials (BSMs) of allogeneic, xenogeneic, or alloplastic origin represent a valid therapeutic option for regenerative therapy after maxillofacial bone loss [1]. However, due to regulatory reasons, all BSMs are processed non-cellularly and therefore contain only osteoconductive properties, whereas autologous bone (with no antigenic properties) is loaded with cells and growth factors that stimulate, inter alia, new blood vessel formation and trigger osteoinduction [2]. In general, a sufficient blood vessel supply and the formation of new blood vessels from pre-existing ones (angiogenesis) are obligatory prerequisite for bony regeneration [3]. Here, homeostasis, structural pathways, and paracrine functions are some of the main features that couple angiogenesis with osteogenesis, especially in the initial and early regenerative phases [4].

However, the translation of tissue and bone engineering methods that enhances the angiogenic properties of BSMs in clinical workflow is limited, mainly due to regulatory reasons. In contrast, autologous platelet concentrates such as platelet-rich fibrin (PRF) that are now broadly used in dental and craniomaxillofacial regenerative medicine may potentially overcome this limitation [5]. Thus far, the pro-angiogenic effect of the PRF has been demonstrated to mainly impact soft tissue regeneration procedures [6,7], but the complex interplay of different cytokines and growth factors leads to an increased proliferation and differentiation of different cell lines, inter alia, osteoblasts [8,9]. In detail, vascular endothelial growth factor (VEGF), tissue growth factor ß (TGFß), and platelet-derived growth factor (PDGF) have been discussed to trigger vasoformative responses [10]. It was evaluated if the combination of PRF with BSMs in different clinical approaches not only functions as a signaling protein reservoir for osteoinduction but also allows the bone graft particles to stick together for better clinical handling [11]. There are emerging data that suggest that this method seems feasible in maxillary sinus floor lift, graft, and surgical augmentation procedures [12]; for guided bone regeneration methods in dental implantology [13]; alveolar ridge preservation [14]; and the treatment of intrabony periodontal defects [15].

However, there is currently no consistent evidence based on basic research that PRF can support osteogenesis [8,16,17]. The ambivalent data may be explained in the different biophysical properties of BSMs and the variety of investigated time points. More basic research is needed to deliver scientific evidence that can be translated into clinical workflow [12].

Recently, our working group showed a positive effect of PRF in combination with allogeneic and xenogeneic BSMs that enhanced osteoblast activity compared to a native BSM in vitro at later time points [7]. Thus far, no comparative study has analyzed the early angiogenetic interactions of PRF with different BSMs in vitro nor translated these results to in vivo experiments.

Therefore, the aim of this study was to assess the underlying initial cellular mechanism in order to validate possible clinical application of this approach. Here, differences in the interactions and activation of platelets of BSMs biofunctionalized with PRF were analyzed, and the possible implication for angiogenesis and neovascularization in vivo were investigated.

## 2. Materials and Methods

### 2.1. Bone Substitute Materials

BSMs of allogeneic (AKM: maxgraft^®^, botiss biomaterials GmbH, Zossen, Germany, granularity < 2 mm), alloplastic (APKM: maxresob^®^, botiss biomaterials GmbH, Zossen, Germany, granularity 0.8–1.5 mm), and xenogenic (XKM1: cerabone^®^, botiss biomaterials GmbH, Zossen, Germany, granularity 1.0–2.0 mm; XKM2: BioOss^®^, Geistlich Pharma AG, Wolhusen, Switzerland, granularity 1–2 mm) origin were analyzed.

### 2.2. PRF Protocol

In accordance with the ethical standards of the national research committee (Ärztekammer Rheinland-Pfalz, no. “2019-14705_1”), 10 mL of peripheral venous blood per sample were collected from three healthy donors without severe illnesses after the puncturing of the cephalic or the median cubital vein. A vacutainer system and specific sterile plain vacuum tubes with additional silicone within their coating surface were, respectively, used for solid (A-PRF+, Mectron, Carasco, Italy) and liquid PRF (iPRF, Mectron, Carasco, Italy). PRF was directly manufactured with a fixed angle rotor with a radius of 110 mm at 1200 rpm and a relative centrifugal force of 177 g for 8 min (Duo centrifuge, Mectron, Carasco, Italy) following the manufacturer’s instructions [7].

### 2.3. Early Interaction of PRF and BSM

To analyze the platelet interaction of BSMs in combination with PRF, 0.5 mL of liquid PRF was transferred into a sterile 5 mL Eppendorf tube (Merck, Darmstadt, Germany), and 100 mg of a native BSM were added (three samples, each in in triplet; total *n* = 54). Afterwards, the samples were gently mixed using a rotator at 15 rpm at room temperature for 15 min in order to obtain an optimal contact between BSMs and PRF, as previously described for platelet-rich plasma (PRP) [18]. One sample with EDTA-blood and one with PRF without any BSMs served as controls, and 20 μL of each sample were used to count the number of remaining non-aggregated platelets using a hematology analyzer (KX21, Sysmex Europe GmbH, Norderstedt, Germany). Afterwards, the supernatant was pipetted off and snap frozen at −80 °C for further investigation.

### 2.4. ELISA Quantification of Early Interaction of PRF and BSM

Growth factor release on a protein basis was evaluated after 15 min of incubation of BSM/PRF in comparison to PRF alone (five samples, each in triplet per antibody; total *n* = 60). Samples were analyzed via an ELISA for VEGF, TGFß, and PDGF (all: R&D Systems, Minneapolis, MN, USA) using the manufacturer’s protocol, as previously described [7]. Measurements were conducted with an ELISA plate reader at 450 nm (Molecular Devices, San Jose, CA, USA) and analyzed using the SoftMax Pro 5.4 (Molecular Devices, San Jose, CA, USA) software.

### 2.5. Scanning Electron Microscopy

To visualize the direct contact of the BSMs with PRF, one sample of each material was prepared for SEM (*n* = 4) in a 24-well plate, as previously described [19]. In brief, after 15 min of incubation, cells were fixed with formaldehyde, dehydrated and mounted on conductive stubs before an SCD 040 sputter-coater (BAL-TEC AG, Leica Microsystems, Solms, Germany) was used to coat samples with gold. Next, SEM micrographs were performed (Philips XL30, Eindhoven, The Netherlands), and images were exploratively analyzed with an analysis program (Kontron KS 300, Carl Zeiss Vision, Eching, Germany).

### 2.6. Quantification of Angiogenesis In Vivo

**Figure 1:**
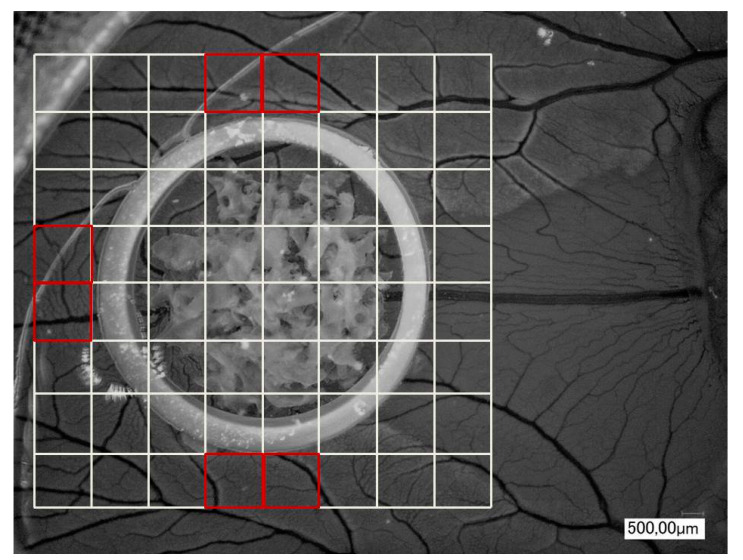
Exemplary picture (30 × magnification) of an incubated bone substitute materials (BSMs) in the chorioallantoic membrane (CAM) assay with the six defined regions of interest per mm^2^ around the ring.

To evaluate the influence of BSM/PRF on angiogenesis in vivo, a chorioallantoic membrane (CAM) assay was used, as previously described [7,20]. The samples, both native (AKM, APKM, XMK1, and XKM2) and with PRF bio-activated BSMs (AKM+, APKM+, XKM1+, and XKM2+), were assessed and compared to the control of the CAM alone (Ctrl.), as well as the pure PRF (triplets per sample; total *n* = 36). In brief, fertilized white Leghorn chicken eggs (LSL Rhein-Main, Dieburg, Germany) were incubated at 38 °C at constant humidity until the fourth day of embryological development. Then, 8–10 mL of egg white were removed with a sterile syringe, and a 3  ×  3 cm^2^ window was cut into the eggshell under sterile conditions. After another 24 h, we applied a sterile orthodontic elastic rubber ring (Elastics, Dentaurum, Ispringen, Germany), into which the samples were inserted. After 24 h of incubation, analysis in 30-fold and 50-fold magnification by centering the ring was performed using a digital microscope (KEYENCE, Neu-Isenburg, Germany) and its software (KEYENCE, Neu-Isenburg, Germany) after overlaying a grid (with a 500 μm side length) over the micrographs and manually counting all vessels and branching points of the vessels in six defined regions of interest per mm^2^ around the ring (Figure 1). Afterwards, the embryos were euthanized by cutting the main vessels.

### 2.7. Immune-Histochemically Display of Vessel Formation

**Figure 2:**
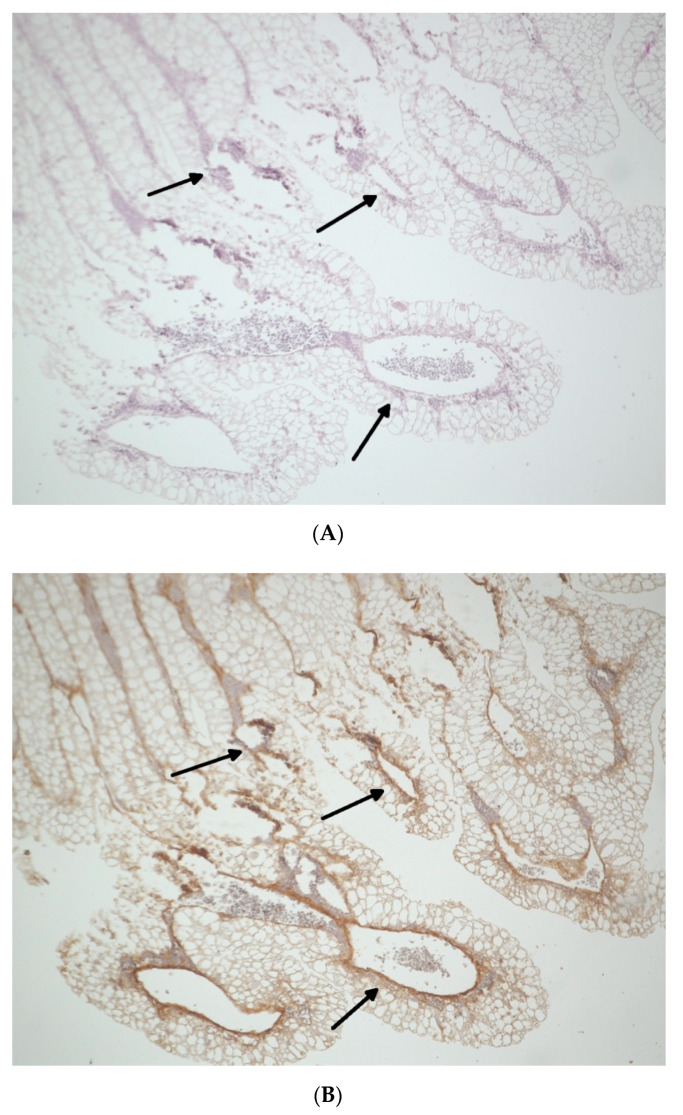
Exemplary samples of the CAM (2× magnification) after incubation of allogeneic BSMs with platelet-rich fibrin (PRF) for 24 h displayed immunohistochemically via hematoxylin–eosin (HE) (**A**) and smooth muscle actin (SMA) (**B**) antibodies. Arrows indicate vessel formation.

For the descriptive immune-histochemical demonstration of the vessels, the ring and each BSM were removed, and the underlying CAM was fixed in formaldehyde for 24 h, embedded in paraffin, and cut in 5-μm-thick slices (triplets per sample; *n* = 36). Subsequently, hematoxylin–eosin (HE, Merck, Darmstadt, Germany) and α-smooth muscle actin (αSMA, Sigma-Aldrich, St. Louis, MO, USA) staining was performed in accordance with the manufacturer’s instructions and as previously described [7]. Vessel formation was analyzed via light microscopy (KEYENCE, Neu-Isenburg, Germany) by using a BZ-II Analyzer (KEYENCE, Neu-Isenburg, Germany) software) after overlaying a grid (with a 500 μm side length) over the micrographs and manually counting all vessels in six defined regions of interest per mm^2^ around the former region of the ring (in Figure 2A,B, arrows mark newly formed vessels).

### 2.8. Statistical Analysis

All results were evaluated in mean values with their standard errors and illustrated as bar charts with error bars. Differences between all groups were analyzed with Kruskal–Wallis rank sum test. After checking on normal distribution with the Shapiro–Wilk test, a Student’s t-test for paired samples (in case of normally distributed values) or a Mann–Whitney test (for non-normal distributions) was used to check for statistically significances (a *p*-value of ≤0.05 was applied).

## 3. Results

### 3.1. Initial Cell–Cell Interaction of BSM in Combination with PRF

**Figure 3:**
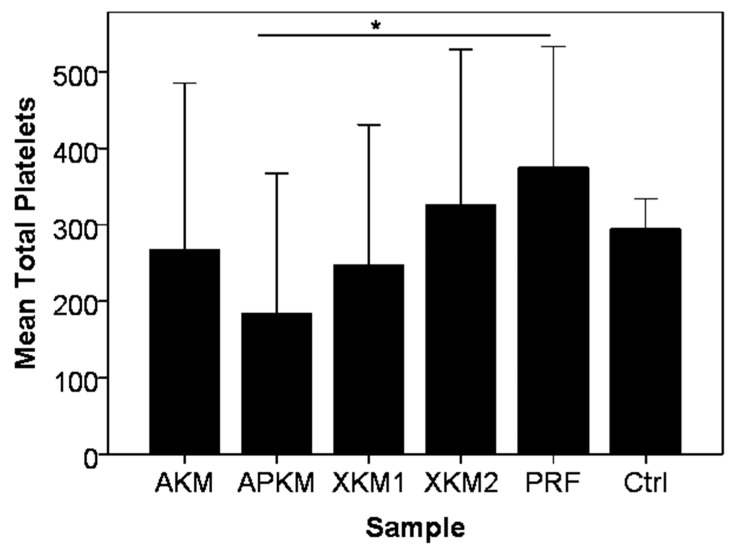
Total platelet count × 10^3^/µL after 15 min of incubation of each BSM with PRF in comparison to PRF alone and EDTA blood (Ctrl: control). In comparison to PRF alone, all BSMs led to an active platelet consumption, with a significant difference for APKM vs. PRF (* *p* = 0.05).

PRF alone showed a higher mean platelet count × 10^3^/µL than whole blood without reaching any reaching statistical significance (*p* = 0.161). The incubation of PRF with all tested BSMs led to a decrease of platelets (AKM: *p* = 0.340; XKM1: *p* = 0.161; and XKM2: *p* = 0.796), with APKM showing the strongest decrease in comparison to PRF alone (*p* = 0.05; Table 1 and Figure 3).

**Figure 4:**
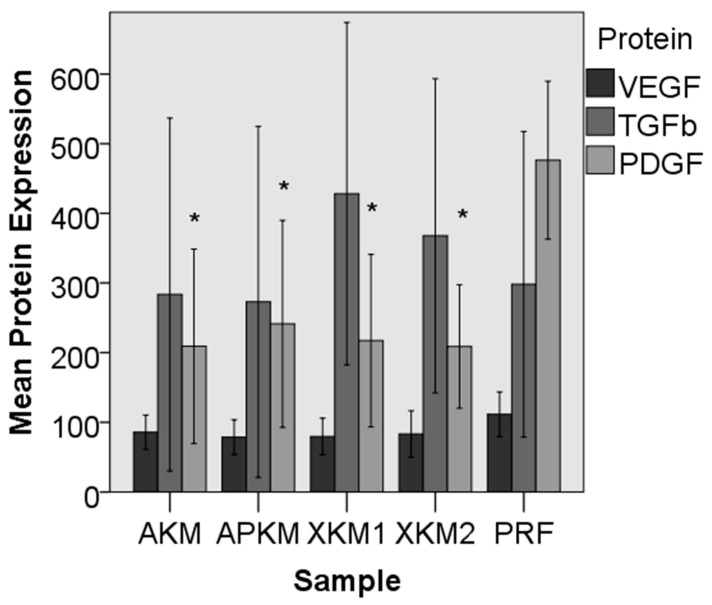
Expression of VEGF, TGF, and PDGF in the BSM samples incubated with PRF in comparison to PRF alone. PDGF was statistically significantly decreased for all tested BSMs in comparison to PRF alone (* *p* < 0.05; for detailed *p*-values, please refer to the text).

In the ELISA experiment, the combination of BSMs with PRF led to a slightly decreased expression of VEGF when compared to PRF alone (AKM: *p* = 0.161; APKM: *p* = 0.089; XKM1: *p* = 0.098; and XKM2: *p* = 0.174). Between the groups, no significant differences could be detected (*p* = 0.35). For TGFß, both xenogeneic BSMs (XKM1 and XKM2) showed an increased expression in comparison to PRF alone but also failed to reach statistical significance (AKM: *p* = 0.512; APKM: *p* = 0.775; XKM1: *p* = 0.285; and XKM2: *p* = 0.838). Furthermore, no significant differences between the samples could be seen (*p* = 0.48). For PDGF, a significantly decreased expression in comparison to PRF alone was shown for all BSMs (AKM: *p* = 0.003; APKM: *p* = 0.002; XKM1: *p* = 0.004; and XKM2: *p* = 0.013). The differences between the groups were statistically significant (*p* = 0.009; Table 2A–C and Figure 4).

**Figure 5:**
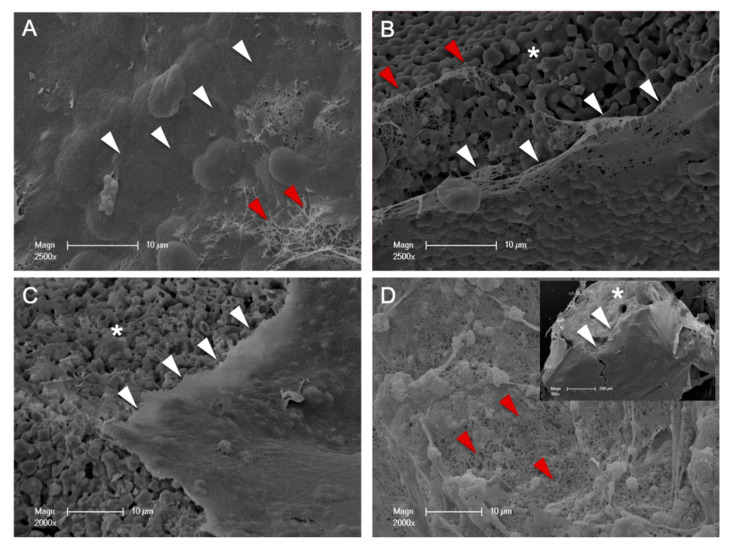
SEM: allogeneic (**A**), alloplastic (**B**), xenogenic material 1 (**C**), and xenogeneic material 2 (**D**) with PRF-biofunctionalization. The surfaces of the bone substitutes (*) were covered by a thin PRF-layer (white arrows) that was created by closely networked fibrin fibers (red arrows).

SEM micrographs showed the BSM surfaces narrowly covered with a thin PRF-layer that is created by closely networked fibrin fibers demonstrated the closest spatial relationship between BSMs and PRF (Figure 5).

### 3.2. Influence of PRF in Combination with Different BSMs on Angiogenesis In Vivo

**Figure 6:**
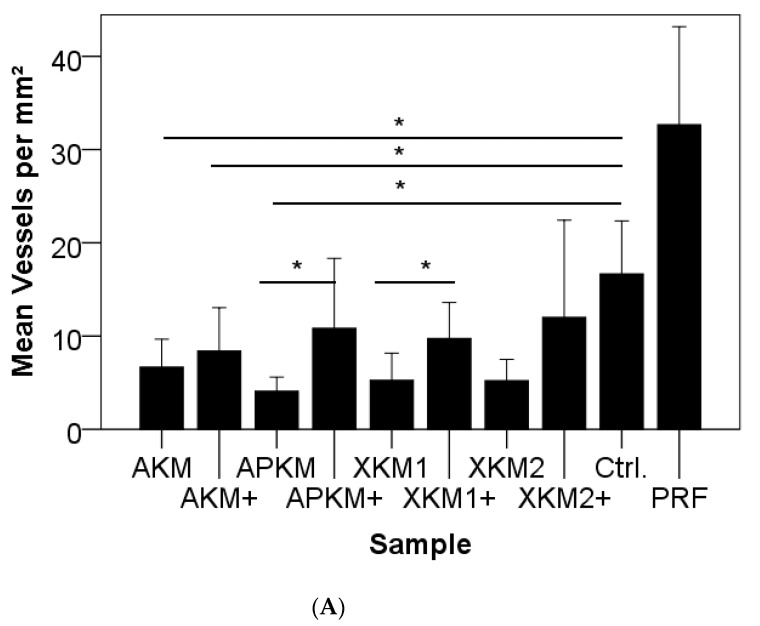

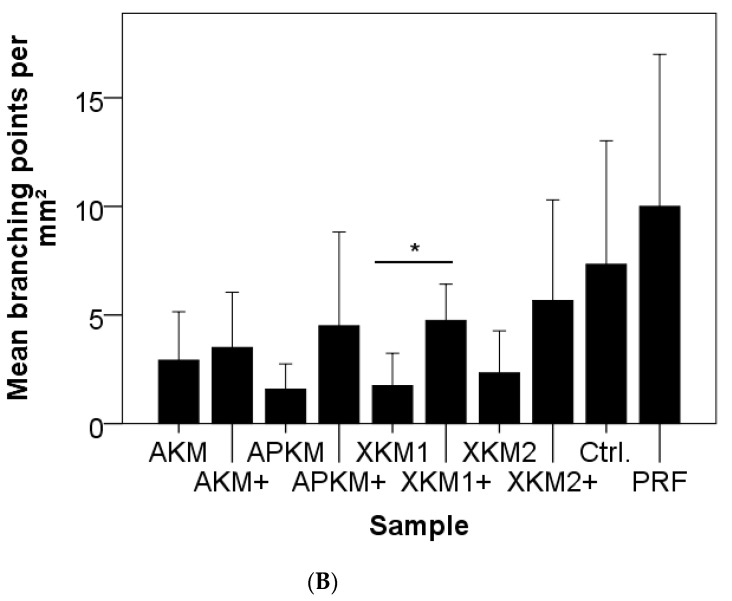
Newly formed vessels per mm^2^ (**A**) and branching points per mm^2^ (**B**) of the CAM after 24 h of incubation of BSMs with (+)/without PRF in comparison to the negative control and PRF alone (* *p* < 0.05).

After 24 h of incubation, the total number of vessels did reveal strong differences between the compared groups (*p* = 0.009). Even though no statistical significance was reached, the total number of vessels was higher for PRF alone in comparison to the negative control group (*p* = 0.127; Figure 6A and Table 3A). In comparison to the negative control, vessel formation was found to be decreased for all tested materials, to a greater extent for native BSMs (AKM: *p* = 0.014; AKM + PRF: *p* = 0.041; APKM: *p* = 0.009; APKM + PRF: *p* = 0.241; XKM1: *p* = 0.018; XKM1 + PRF: *p* = 0.051; XKM2: *p* = 0.012; and XKM2 + PRF: *p* = 0.302). Accordingly, the BSMs in combination with PRF did show significant positive pro-angiogenic effects in comparison to their native correspondents for alloplastic and xenogeneic materials (AKM vs. AKM+: *p* = 0.406; APKM vs. APKM+: *p* = 0.007; XKM1 vs. XKM1+: *p* = 0.015; and XKM2 vs. XKM2+: *p* = 0.120).

For the evaluation of the branching points per mm^2^, no significant differences between groups was found (*p* = 0.15). Though PRF alone increased branching points in comparison to the negative control, the difference was not statistically significant (*p* = 0.275). In comparison to the negative control, vessel formation was found to be decreased for all tested materials, to a greater extent for native BSMs (AKM: *p* = 0.217; AKM + PRF: *p* = 0.347; APKM: *p* = 0.138; APKM + PRF: *p* = 0.510; XKM1: *p* = 0.133; XKM1 + PRF: *p* = 0.412; XKM2: *p* = 0.211; and XKM2 + PRF: *p* = 0.696). A tendency for a higher number of branching points for was detected for bio-activated BSMs, but only one xenogeneic material reached statistical significance (AKM vs. AKM + PRF+: *p* = 0.688; APKM vs. APKM + PRF: *p* = 0.135; XKM1 vs. XKM1 + PRF: *p* = 0.005; and XKM2 vs. XKM2 + PRF: *p* = 0.103; Figure 6B and Table 3B).

**Figure 7:**
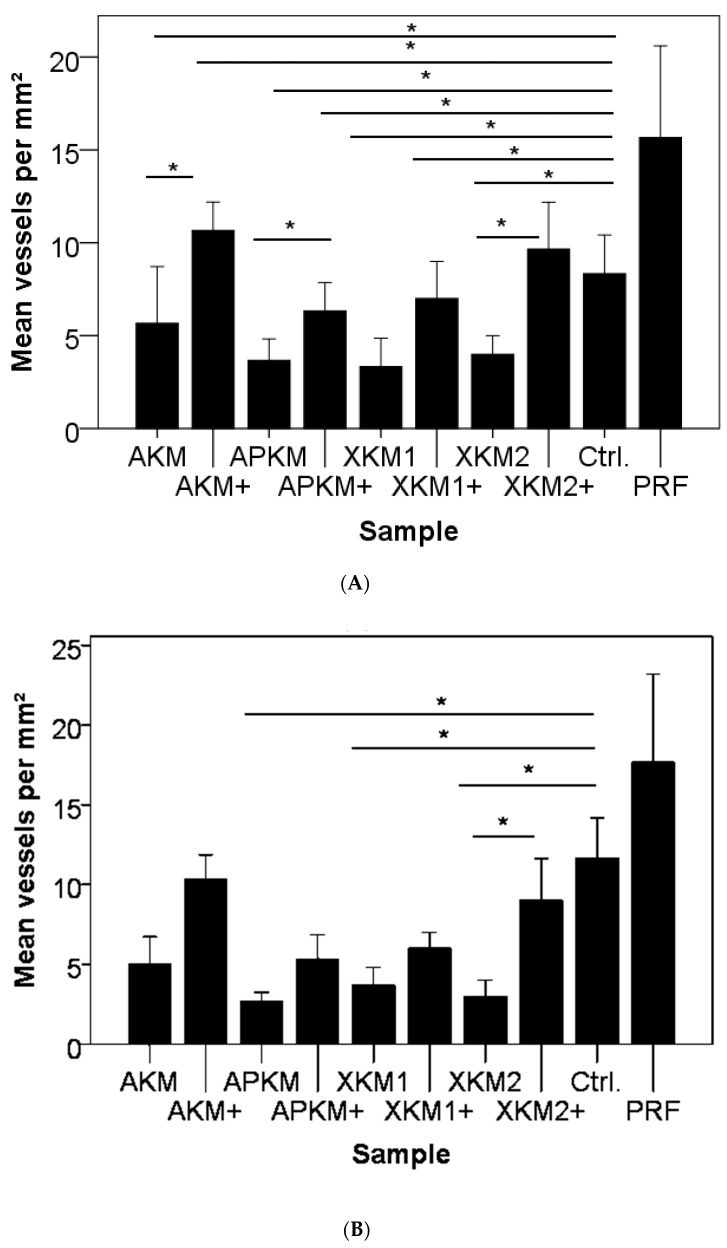
Newly formed vessels per mm^2^ after HE (**A**) and after αSMA staining (**B**) after the removal of each BSM with (+)/without PRF in comparison to the negative control and PRF alone (* *p* < 0.05).

To visualize newly formed vessels, immune-histochemical staining for HE and αSMA was performed, as indicated in representative micrographs (triplets per sample; *n* = 36). For HE (Figure 7A and Table 4A) and αSMA staining (Figure 7B and Table 4B), no significant differences between the groups were found (*p* = 0.15). Furthermore, PRF alone did not significantly increase vessel amount in comparison to the negative control (HE: *p* = 0.184; αSMA: *p* = 0.077). After HE staining, BSMs with or without PRF had decreases in new vessels in comparison to the negative control (AKM: *p* = 0.046; AKM + PRF: *p* = 0.500; APKM: *p* = 0.046; APKM + PRF: *p* = 0.050; XKM1: *p* = 0.046; XKM1 + PRF: *p* = 0.050; XKM2: *p* = 0.050; and XKM2 + PRF: *p* = 0.184). In contrast, vessel formation was significantly increased when the bio-activated allogeneic, alloplastic, and xenogeneic BSMs were compared to the native control except for XKM1 (AKM vs. AKM + PRF: *p* = 0.046; APKM vs. APKM + PRF: *p* = 0.046; XKM1 vs. XKM1 + PRF: *p* = 0.072; and XKM2 vs. XKM2 + PRF: *p* = 0.05). After αSMA staining, native BSMs displayed less vessels in comparison to the negative control (AKM: *p* = 0.184; AKM+ PRF: *p* = 0.184; APKM: *p* = 0.046; APKM + PRF: *p* = 0.184; XKM1: *p* = 0.050; XKM1 + PRF: *p* = 0.376; XKM2: *p* = 0.050; and XKM2 + PRF: *p* = 0.376). In contrast, vessel formation was significantly increased when the bio-activated allogeneic and alloplastic BSMs, as well as XKM2, were compared to the native control (AKM vs. AKM + PRF: *p* = 0.077; APKM vs. APKM + PRF: *p* = 0.072; XKM1 vs. XKM1 + PRF: *p* = 0.077; and XKM2 vs. XKM2 + PRF: *p* = 0.050).

## 4. Discussion

As a basic research study, this work presents a comparative in vitro analysis of the initial angiogenic interaction and subsequent growth factor expression pattern of BSMs of different origins in combination with PRF. In addition, a CAM assay was used to evaluate possible implications for early angiogenesis and new vessel formation in vivo. In this way, scientific evidence for the clinical and surgical use of PRF as an effective additive to BSMs was analyzed. Among the main findings, the combination of BSMs with PRF led to an initial platelet consumption with a significant decrease of the growth factor expression of PDGF into the supernatant after 15 min. Since platelet count decreases with correlation to platelet consumption [18], it can be hypothesized that PRF initially interacts with its respective BSM via platelet activation. Here, the alloplastic BSM showed the strongest decrease, thus indicating the strongest interaction. To the best of our knowledge, there has not been any other study that has analyzed this possible implication. However, studies with other platelet concentrates such as PRP have revealed similar results [18]. In contrast to the literature [18,21], ELISA quantification has revealed a decrease of VEGF and especially PDGF for all BSMs in this study. Only TGFß, mainly for xenogeneic materials, was slightly increased in comparison to PRF alone. A possible explanation for this observation may be related to a specific cytokine retention by physical interaction with BSMs of different surfaces [18]. It is known that the growth factor release of PRF alone follows certain dynamics with a peak between three and seven days that triggers the migration of different cell lines such as human umbilical vein endothelial cells at these time points and, therefore, directly optimizes vessel formation [22]. However, analyses of these kinetics in combination with BSMs or other biomaterials are sparse. A study by Castro et al. did show a continuous growth factor release from the PRF in combination with a xenogeneic scaffold for 14 days. Interestingly, the authors found a lower extent of growth factor release for a PRF/xenograft combination in comparison to PRF alone, similar to the results presented in this study. They concluded that the PRF became physically entrapped within the xenograft, and release occurred passively as the close fibrin network was degraded via the serin protease plasmin [23].

In this context, the SEM micrographs presented in this study demonstrated a close fibrin network of the PRF, directly in contact with the tested BSMs, that may have contributed to the “storage” of the growth factors within the BSMs. Analogously, a recent study by our working group found that PRF in combination with allogeneic and xenogeneic BSMs enhances osteoblast activity for up to 10 days in vitro [24]. In addition to BSM-induced platelet activation and degranulation, simultaneous cytokine retention with a consecutive and consistent slow release over a physiologic time period in physiologic levels may lead to the these results and, finally, successful tissue regeneration approaches [18].

In general, with recent adjustments in regulatory requirements by the Food and Drug Administration, the application of growth factors related to bone substitute materials, such as recombinant human bone morphogenetic proteins (rhBMPs) [25], platelet-derived growth factor-BB (rhPDGF) [26], and fibroblast growth factor-2 (rhFGF-2) [27], has emerged as a new frontier in the field of reconstructive surgery. Currently, there is an ongoing debate on appropriate concentrations and indications [28]. Interestingly, an analysis of gingival fluid growth factor levels during early the healing period after regeneration of intrabony defects with tricalcium phosphate and PRF vs. a collagen membrane containing rhPDGF showed “similar early wound healing outcomes, although the levels of PDGF were higher in the PRF membrane group for [an] extended period of time“ in a recent clinical trial. The authors stated PRF to be a biodegradable and inexpensive growth factor eluting guided tissue membrane regeneration [29]. Therefore, one may hypothesize that despite the decreased growth factor levels found after the interaction of PRF with each BSM, a significant level could be reached in cervical fluid in clinical applications.

Furthermore, possible implications for angiogenesis were analyzed via the CAM assay in vivo. The assay has been well-described for this indication and widely used in the literature [7,20]. As a major advantage, this approach is in accordance with the concept of the replacement of experimental animals, the reduction of the total number of experimental animals needed, and refined testing protocols (“3R aspects”: replacement, reduction, refinement [30]). In addition, it can respond to osteogenic stimuli and offers significant potential as an in vivo model for xenograft organ culture [31,32]. Evidence has shown that the assay may also be used to evaluate possible inflammatory processes and is therefore of great interest for future studies in the field of tissue engineering, especially with platelet-derived concentrates [20,33]. Here, microscopically and immune-histochemically, all tested BSMs led to a noteworthy decrease in vessel-formation and branching-points in comparison to PRF alone. However, PRF had a significant positive pro-angiogenic effect, especially in combination with alloplastic and xenogeneic materials, thus strengthening the above-mentioned hypothesis of the consecutive release of growth factors through the fibrin network that triggers vasoformative responses. In this context, Ratajczak et al. evaluated the angiogenic capacity of PRF in a CAM assay and found induced blood vessel formation [34]. Thus far, studies that have tested bone substitutes with the CAM assay are sparse [33,35], and no analysis that has evaluated PRF combined with BSMs has been found. Recently, our working group demonstrated that the biofunctionalization of collagen matrices with PRF led to an increased angiogenic potential, as evaluated by the CAM assay. In this study, similar to the presented approach, the matrix was incubated with PRF for only 24 h [7]. This strengthened the hypothesis that PRF may influence the angiogenic potential of different scaffolds at this early time point. The consecutive release of the stored cytokine within the biofunctionalized biomaterial then may improve long-term tissue integration and regeneration. This is in accordance with other in vivo studies. In a murine model, PRF alone did not enhance bone regeneration in non-critical size defects and even had a temporary negative influence on RUNX and VEGF expression [36]. However, combined with an alloplastic hydroxyapatite and ß-tricalcium phosphate, a positive effect on bone formation was found [37]. For other xenogeneic materials, the combination of PRF improved bone repair [38,39,40]. For allografts with PRF, a randomized clinical trial found the combination effective in alveolar ridge preservation [41].

This study suffered from some major limitations. First, the in vitro approach could not describe complex biological interactions occurring in an organism in toto due to the lack of systemic and local factors arising from the complexity of cell and tissue responses [42]. Secondly, only a small sample size was achieved, which led to statistical interpretations of the results that were worth considering. However, the reliability in vitro testing for preclinical biomaterial evaluation is increasingly improving through the continuous expansions of knowledge from basic research, adjustments of in vitro systems, “3R aspects,” and comparative science between humans and animals. Systematic reviews of animal or in vitro research, if they are used to inform the design of clinical trials (particularly with respect to appropriate drug dose, timing, and other crucial aspects of the drug regimen), will further improve the predictability of animal research in human clinical trials.

Within the limitations of this study, no recommendation can be given regarding which BSM may most interact with PRF to optimize bony regeneration. However, it seems that alloplastic and xenogeneic materials may benefit the intended pro-angiogenic effect at an early time point to the greatest extents. This needs to be further addressed to support clinician scientists and surgeons in the field.

## 5. Conclusions

To conclude, we demonstrated an initial cell–cell interaction of PRF and different BSMs that led to noteworthy changes in growth factor expression in vitro and angiogenic features in vivo. This work provides clinician scientists and surgeons with scientific evidence for the use of PRF as a possible additive to BSMs in surgical reconstruction. Therefore, the presented study may help to translate this practicable method to trigger combined soft and hard tissue defects in the clinical workflow.

## Figures and Tables

**Table 1:** Mean total platelet count x 10^3^/µL with standard error after 15 min of incubation of each BSM with PRF in comparison to PRF alone and EDTA blood. *p*-values are given for the comparison of each sample with PRF alone (Mann–Whitney U-test).

Sample	Mean Platelet x 10^3^/µL	*p*-Value (Sample vs. PRF, Mann–Whitney U Test)
PRF	374.66 ± 158.16	-
Blood	294.44 ± 39.83	0.161
AKM	267.22 ± 218.23	0.340
APKM	183.66 ± 183.06	0.05
XKM1	246.67 ± 184.06	0.161
XKM2	326.44 ± 202.59	0.796

**Table 2:** Mean protein expression via ELISA for (**A**) vascular endothelial growth factor (VEGF), (**B**) tissue growth factor ß (TGFß) and (**C**) platelet-derived growth factor (PDGF) in pg/mL with standard error after 15 min of incubation of each BSM with PRF in comparison to PRF alone. *p*-values are given for the comparison of each sample with PRF alone (Mann–Whitney U test).

**A**		
**Sample**	**Mean VEGF Expression (pg/mL)**	***p*-Value (Sample vs. PRF, Mann–Whitney U Test)**
PRF	111.47 ± 58.04	-
AKM	85.90 ± 44.27	0.161
APKM	78.75 ± 45.00	0.089
XKM1	79.68 ± 47.48	0.098
XKM2	83.34 ± 59.95	0.174
**B**		
**Sample**	**Mean TGFß Expression (pg/mL)**	***p*-Value (Sample vs. PRF, Mann–Whitney U Test)**
PRF	298.16 ± 396.15	-
AKM	283.44 ± 457.51	0.512
APKM	272.95 ± 455.23	0.775
XKM1	428.20 ± 444.10	0.285
XKM2	367.81 ± 407.39	0.838
**C**		
**Sample**	**Mean PDGF Expression (pg/mL)**	***p*-Value (Sample vs. PRF, Mann–Whitney U Test)**
PRF	476.29 ± 204.94	-
AKM	209.08 ± 252.08	0.003
APKM	241.29 ± 268.21	0.002
XKM1	217.12 ± 223.60	0.004
XKM2	208.82 ± 160.05	0.013

**Table 3:** Mean vessels/mm^2^ (**A**) and branching points per mm^2^ (**B**) after 24 h of incubation of each sample within the CAM assay in comparison to the negative control and PRF alone, evaluated via light microscopy. *p*-values are given for a comparison of each sample with the control and with their native BSM (Mann–Whitney U test).

**A**			
**Sample**	**Mean Number of Vessels**	***p*** **-Value (Sample vs. Control, Mann–Whitney U Test)**	***p*** **-Value (Sample vs. Native BSM, Mann–Whitney U Test)**
Control	16.67 ± 5.68	-	-
PRF	32.66 ± 10.50	0.127	-
AKM	6.66 ± 2.99	0.014	*p* = 0.406
AKM+	8.40 ± 4.64	0.041	
APKM	4.08 ± 1.50	0.009	*p* = 0.007
APKM+	10.83 ± 7.49	0.241	
XKM1	5.25 ± 2.915	0.018	*p* = 0.015
XKM1+	9.75 ± 3.84	0.051	
XKM2	5.22 ± 2.27	0.012	*p* = 0.120
XKM2+	12.00 ± 10.43	0.302	
**B**			
**Sample**	**Mean Number of Branching Points/mm^2^**	***p*** **-Value (Sample vs. Control, Mann–Whitney U Test)**	***p*** **-Value (Sample vs. Native BSM, Mann–Whitney U Test)**
Control	7.33 ± 5.68	-	-
PRF	10.00 ± 7.00	0.275	-
AKM	2.91 ± 2.23	0.217	*p* = 0.688
AKM+	3.50 ± 2.54	0.347	
APKM	1.58 ± 1.16	0.138	*p* = 0.135
APKM+	4.5 ± 4.32	0.510	
XKM1	1.75 ± 1.48	0.133	*p* = 0.005
XKM1+	4.75 ± 1.66	0.412	
XKM2	2.33 ± 1.93	0.211	*p* = 0.103
XKM2+	5.66 ± 4.63	0.696	

**Table 4:** Mean vessels/mm^2^ after HE staining (**A**) and aSMA staining (**B**) after 24 h of incubation of each sample within the CAM assay in comparison to the negative control and PRF alone, evaluated immune-histochemically. *p*-values are given for a comparison of each sample with the control and with their native BSM (Mann–Whitney U test).

**A**			
**Sample**	**Mean Number of Vessels/mm^2^ (HE Staining)**	***p*-Value (Sample vs. Control, Mann–Whitney U Test)**	***p*-Value (Sample vs. Native BSM, Mann–Whitney U Test)**
Control	11.66 ± 2.51	-	-
PRF	17.66 ± 5.50	0.200	-
AKM	5 ± 1.73	0.046	*p* = 0.046
AKM+	10.33 ± 1.52	0.500	
APKM	2.66 ± 0.57	0.046	*p* = 0.046
APKM+	5.33 ± 1.52	0.050	
XKM1	3.66 ± 1.15	0.046	*p* = 0.072
XKM1+	6 ± 1.00	0.050	
XKM2	3.00 ± 1.00	0.050	*p* = 0.050
XKM2+	9.00 ± 2.64	0.184	
**B**			
**Sample**	**Mean Number of Vessels/mm^2^ (aSMA Staining)**	***p*-Value (Sample vs. Control, Mann–Whitney U Test)**	***p*-Value (Sample vs. Native BSM, Mann–Whitney U Test)**
Control	8.33 ± 2.08	-	-
PRF	15.66 ± 4.93	0.077	-
AKM	5.66 ± 3.05	0.184	*p* = 0.077
AKM+	10.66 ± 1.53	0.184	
APKM	3.66 ± 1.15	0.046	*p* = 0.072
APKM+	6.33 ± 1.52	0.184	
XKM1	3.33 ± 1.15	0.050	*p* = 0.077
XKM1+	7.00 ± 2.00	0.376	
XKM2	4.00 ± 1.00	0.050	*p* = 0.050
XKM2+	9.66 ± 2.51	0.376	

## Data Availability

All data is displayed in the manuscript.
